# Scoping Review on ACL Surgery and Registry Data

**DOI:** 10.1007/s12178-022-09775-2

**Published:** 2022-07-13

**Authors:** Janina Kaarre, Bálint Zsidai, Eric Narup, Alexandra Horvath, Eleonor Svantesson, Eric Hamrin Senorski, Alberto Grassi, Volker Musahl, Kristian Samuelsson

**Affiliations:** 1 Department of Orthopaedics, Institute of Clinical Sciences, Sahlgrenska Academy, University of Gothenburg, Gothenburg, Sweden; 2 Sahlgrenska Sports Medicine Center (SSMC), Gothenburg, Sweden; 3 Department of Orthopaedic Surgery, UPMC Freddie Fu Sports Medicine Center, University of Pittsburgh, Pittsburgh, PA USA; 4 Department of Internal Medicine and Clinical Nutrition, Institute of Medicine, Sahlgrenska Academy, University of Gothenburg, Gothenburg, Sweden; 5 Unit of Physiotherapy, Department of Health and Rehabilitation, Institute of Neuroscience and Physiology, Sahlgrenska Academy, University of Gothenburg, Gothenburg, Sweden; 6 IIa Clinica Ortopedica e Traumatologica, IRCCS Istituto Ortopedico Rizzoli, Bologna, Italy; 7 Department of Orthopaedics, Sahlgrenska University Hospital, Mölndal, Sweden

**Keywords:** Registry data, Anterior cruciate ligament, Anterior cruciate ligament reconstruction, Registry-based research, Registry

## Abstract

**Purpose of Review:**

To present an overview of registry-based anterior cruciate ligament (ACL) research, as well as provide insight into the future of ACL registries.

**Recent Findings:**

During the past decades, the ACL registries have had an important role in increasing our understanding of patients with ACL injuries and their treatment. The registry data has deepened our understanding of factors that have been associated with an increased risk of sustaining an ACL injury and for evaluation of treatment factors and their impact on patient-related outcomes. Recently, registry-based ACL research using artificial intelligence (AI) and machine learning (ML) has shown potential to create clinical decision-making tools and analyzing outcomes. Thus, standardization of collected data between the registries is needed to facilitate the further collaboration between registries and to facilitate the interpretation of results and subsequently improve the possibilities for implementation of AI and ML in the registry-based research.

**Summary:**

Several studies have been based on the current ACL registries providing an insight into the epidemiology of ACL injuries as well as outcomes following ACL reconstruction. However, the current ACL registries are facing future challenges, and thus, new methods and techniques are needed to ensure further good quality and clinical applicability of study findings based on ACL registry data.

## Introduction

Injury to the anterior cruciate ligament (ACL) is a common musculoskeletal injury potentially leading to severe short- and long-term complications. Although several randomized controlled trials (RCTs) and registry-based studies on patients with ACL injury have been published, the knowledge on the optimal type of treatment, including the timing of surgery, choice of graft type, and treatment of other concomitant injuries, is still lacking. National and regional ACL registries containing a large amount of data have provided the opportunity to increase our understanding of ACL injuries and their treatment [1, 2]. At date, there are several ongoing projects based on various ACL registries aiming to determine how to optimize treatment and pre- and postoperative rehabilitation and improve patient-related outcome measures (PROMs), as well as identifying risk factors for a second ACL injury.

During the past decades, ACL registries have generally provided an increased understanding of the associations between different patient characteristics and patient-reported outcomes as well as the risk of sustaining a second ACL injury. Also, the ACL registries, such as the Scandinavian ACL registries, have contributed to increased knowledge of outcomes following ACL injury, allowing improved understanding of the association between different treatment options and PROMs [3–6]. However, the evidence on how to treat patients that have sustained an ACL injury is still insufficient in terms of timing of surgery, graft type, and treatment type, showing the importance of further studies and continuous development of national ACL registries. Consequently, this review aims to describe both different ACL registries, as well as present different variables and methods used in these registries to provide an overview of registry-based ACL research. Additionally, this current review will discuss the future of ACL research based on registry data and present strengths and limitations associated with registry-based research.

## ACL Registries

**Fig. 1:**
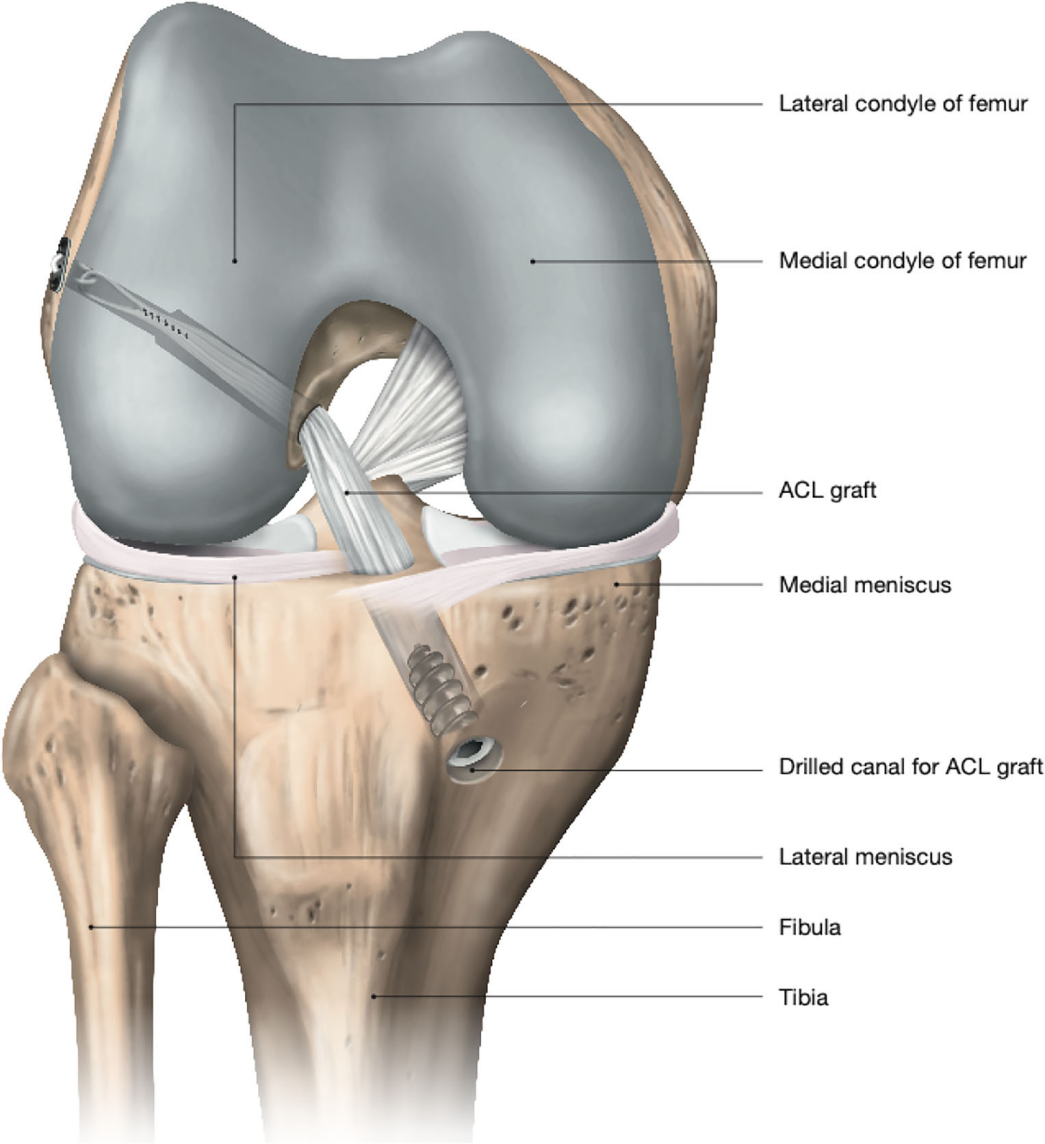
Schematic illustration of the anatomic single-bundle ACL-R technique. *ACL* anterior cruciate ligament, *ACL-R* anterior cruciate ligament reconstruction

In general, the primary purpose of ACL registries is to collect data on patients undergoing ACL reconstruction (ACL-R) (Fig. 1), including information related to patient characteristics, mechanisms of injury, surgical factors, and outcome measures. Some of the ACL registries, such as the Swedish and Luxembourg ACL registries, also include data on non-operatively treated ACL injuries allowing the possibility of comparing outcomes between reconstructive and non-reconstructive treatment of ACL injuries [7, 8]. Furthermore, data related to concomitant injuries, such as injuries to the meniscus, cartilage, and other knee ligaments, are usually collected and included in the ACL registries. Currently, there are seven ACL registers around the world including national ACL registries in Denmark, Luxembourg, New Zealand, Norway, Sweden, United Kingdom (UK), and a regional-based registry in the United States (US) (Table 1). These ACL registries have allowed possibilities to determine appropriate treatment types as well as identifying essential factors for improving treatment of ACL injuries [1]. Furthermore, identification of failures, including revisions and reoperations, and PROMs have provided a possibility for continuous evaluation of clinical practice and thus contributed to improvements in treatment planning of ACL injuries.

**Table 1:** ACL registries

The Danish Knee Ligament Reconstruction Registry	
The Kaiser Permanente ACLR Registry	
The Luxembourg Ligament Register	
The New Zealand ACL Registry	
The Norwegian Knee Ligament Register	
The Swedish National Knee Ligament Register	
The UK National Ligament Register	

*ACL* anterior cruciate ligament, *ACLR* anterior cruciate ligament reconstruction, *the UK* United Kingdom

### The Swedish National Knee Ligament Registry

The Swedish National Knee Ligament Registry (SNKLR) was established in 2005 aiming to collect data on patients undergoing primary ACL-R, revision ACL-R, and reoperations due to other reasons [9]. Today the SNKLR also collects information on non-surgically treated ACL injuries and data on patients sustaining a PCL injury. The registry includes data on patient characteristics (sex, age, body mass index (BMI)), activities at the time of injury (sport, traffic-related activities), surgical factors (graft size, graft type, timing of surgery), and outcome measures (revision, PROMs) [9]. While the responsible surgeon reports all patient- and surgery-related factors, the patient is asked to report PROMs preoperatively and 1, 2, 5, and 10 years postoperatively using a web-based questionnaire. The overall coverage has been previously established to be >90% including data on both private and public healthcare providers [10].

### The Norwegian Knee Ligament Register

The Norwegian Knee Ligament Register (NKLR) was initiated in 2004 with a goal of collecting information on primary and revision ACL and PCL reconstructions [11]. Before the data reporting to the NKLR became mandatory in 2017, the overall coverage of all the ACL injuries in the Norwegian population was reported to be between 84 and 97% [11–13]. The NKLR contains data including patient-related data, surgical data including intraoperative findings, and information regarding subsequent surgeries and PROMs.

### The Danish Knee Ligament Reconstruction Registry

The Danish Knee Ligament Reconstruction Registry (DKRR) was established in 2005 and has been reported to have an estimated coverage ranging from 85 to 92% [14]. The aim of the DKRR is to collect information on epidemiological factors, surgical factors, and PROMs including data on surgical techniques for primary and revision ACL as well as PCL surgeries [14]. Both private and public hospitals are required to submit data in this national registry and the data on preoperative, intraoperative, and 1-year surgical outcome measures are reported by the responsible surgeons.

### The UK National Ligament Register

The UK National Ligament Register (NLR) was started in 2013 with the objective to collect information on ACL injuries, including similar data to the Scandinavian knee ligament registries [15]. Surgeons are asked to submit data on surgical factors as well as any adverse events, while patients are asked to report demographical characteristics, injury-related factors, and outcome measures. The participation is voluntary for all patients with ACL injuries and surgeons.

### The Luxembourg Ligament Register

The Luxembourg Ligament Register was started in 2011 with the purpose to collect data on patients with clinically documented and magnetic resonance imaging (MRI) verified ACL injuries including both operative and non-operative treated ACL injuries [7]. The data is reported both by surgeons and patients, where surgeons are responsible to submit data related to surgery, while patients are asked to report information on demographical as well as injury-related factors by using questionnaires. One-year outcomes including both information on revision surgeries and reoperations, as well as surgeries due to other knee pathologies, are collected. The overall registration rate of ACL injuries to the Luxembourg Ligament Register is estimated to be between 90 and 95% [2].

### The Kaiser Permanente ACLR Registry

The Kaiser Permanente ACLR Registry, located in the USA, is a large region-based ACL registry which was established in 2005. While this registry is not a national registry, the registry includes a large number of patients from an integrated healthcare system with more than 11.2 million people. The registry aims to collect patient-, surgeon-, and healthcare center information data as well as data including surgical factors. In 2010, the voluntary participation was estimated to be around 93% [16].

### New Zealand ACL Registry

The New Zealand ACL Registry (NZACL) was started in 2014 with the purpose to prospectively collect both patient- and surgery-related as well as follow-up data [17]. Since 2017, it has been mandatory for surgeons to report data on patients undergoing ACL-R and the coverage rate has been estimated to be around 85% [17]. While patient-related demographical data is reported by the patient, the operative data including detailed information on ACL-R is reported by the responsible surgeon. Additionally, the complication related to the patient is confirmed by the responsible surgeon.

## Outcome Measures

During the past decades, there has been a growing interest in using outcome measures for evaluating clinical effects in patients undergoing treatment for ACL injuries providing increased understanding of risks and benefits related to different treatment options [18]. Consequently, including data on outcome measures in individual ACL registries has provided the opportunity for continuous clinical evaluation and outcome research [2]. However, there is variation in the use of outcome measures within the individual ACL registries allowing both possibilities for conducting different registry-based studies, but also challenges for combining and comparing data from different registries. Table 2 summarizes the outcome measures used in ACL registries.

**Table 2:** Examples of outcome measures used in ACL registries

Scandinavian ACL registries	Revision rate
	KOOS
	EQ-5D
	Tegner Activity Scale
The Kaiser Permanente ACLR Registry	Revision rate
	KOOS
The Luxembourg Ligament Register	Revision rate
	Reoperation
The New Zealand ACL Registry	Revision rate
	KOOS
	MARS
The UK National Ligament Registry	Revision rate
	KOOS
	Tegner Activity Scale
	IKDC
	EQ-5D

*ACL* anterior cruciate ligament, *ACLR* anterior cruciate ligament reconstruction, *EQ-5D* European Quality of Life Dimension Questionnaire, *IKDC* International Knee Documentation Committee, *KOOS* the Knee Injury and Osteoarthritis Outcome Score, *MARS* Marx Activity Rating Scale, *the UK* United Kingdom

### Outcome Measures in the Scandinavian ACL Registries

Several different outcome measures are included in the Scandinavian ACL registries consisting of information related to the need of later revision surgery and PROMs. The Knee injury and Osteoarthritis Outcome Score (KOOS) has been collected by all the Scandinavian registries. The KOOS is reported and completed by patients preoperatively as well as at 1-, 2-, 5-, or 10-year follow-ups depending on the specific registry [13, 19]. Originally, the KOOS was developed to evaluate both short- and long-term outcomes in patients with osteoarthritis, but has later been validated for other orthopedic approaches including ACL-R [20]. Furthermore, the KOOS consists of five subscales including symptom, pain, activities of daily living, function in sport and recreational, and knee-related quality of life and is scaled from 0 to 100, where a greater score indicates better outcome [21]. Additionally, the SNKLR collects information using the European Quality of Life Five Dimension Questionnaire (EQ-5D), while the DKRR also includes data on the Tegner Activity Scale. The EQ-5D consists of five dimensions including mobility, self-care, usual activities, pain/discomfort, and anxiety/depression and was developed to measure health on a scale of 0 to 1 [22], while the Tegner Activity Score was created to measure the level of activity on a scale of 0 to 10 [23, 24].

### Outcome Measures in Other ACL Registries

More variation regarding the use of outcome measures is found within the non-Scandinavian ACL registries. While the Luxembourg Ligament Register only collects outcome data related to revisions and reoperations, the NLR includes outcome data on PROMs, such as the KOOS, Tegner Activity Scale, International Knee Documentation Committee (IKDC) subjective scores, and EQ-5D. The IKDC, which is one of the most commonly used instruments for evaluating postoperative results following knee surgeries, was designed to be a knee-specific patient-reported outcome measure evaluating patients’ symptoms, sports activity, and knee function on a scale of 0 to 100 [25]. Furthermore, both the Kaiser Permanente Registry and the NZACL also collect outcome data on PROMs including information on KOOS. Additionally, the NZACL collects outcome data based on the Marx Activity Rating Scale (MARS) measuring functional activities associated with high-level knee function (running, cutting, decelerating, and pivoting) on a scale of 0 to 16 [26]. In conclusion, several different PROMs have been used for various research purposes in registry-based studies demonstrating different ways to evaluate outcomes.

## Methods Used in Registry Studies

The foundation of ACL registries in Luxembourg, New Zealand, Scandinavia, the UK, and the USA was followed by an increasing variety of methods harnessed for the analysis and interpretation of demographic, surgical, and outcome data. While some study designs were popularized early on, more robust datasets and innovative statistical approaches have expanded the scope of registry-based ACL research.

### Epidemiology

Surgical ACL registries play an important role in characterizing the prevalence of surgically treated ACL tears at the population level. Early registry studies from Scandinavia have helped establish the frequency of surgically treated isolated and combined ACL tears, concomitant intraarticular injuries, and group-level demographics of patients undergoing ACL surgery [19, 27–29]. Additionally, large-scale epidemiologic studies have enabled the investigation of international differences in variables such as graft choice and surgical timing in ACL-R [30]. An emerging challenge in the domain of registry-based ACL research is to encourage registration of a standardized set of variables across registries, the collection of a more comprehensive list of variables, and the use of relevant, content-valid PROMs for functional outcome assessment [31].

### Comparative Studies

Over the past decade, the majority of ACL registry studies were conducted with the aim to identify differences between treatment variables on the functional outcomes of patients with ACL tears. Investigations with regard to graft choice, surgical techniques, the timing of surgery, and operative versus non-operative treatment are currently some of the most contested topics, with numerous studies trying to identify factors that lead to superior outcomes in specific patient populations.

Historically, the majority of ACL-Rs in Scandinavian countries were performed using hamstring tendon autograft (HT). In contrast, the use of allograft tissue and bone-patellar tendon-bone autograft (BPTB) is more common in the USA. Notably, the increasing popularity of the quadriceps tendon autograft (QT) demonstrates the versatility of available graft choices and the need to clarify which patient groups benefit from ACL-R with a specific graft type. Consequently, the aim of several recent registry studies was to identify differences in revision rate and PROMs when PBTB, HT, QT, and allograft tissue were used for ACL-R [32–34].

Registry studies also enable the comparison of various surgical techniques with a potential impact on failure rate and PROMs following ACL-R. Data from the SNKLR was recently used to compare the frequency of revision ACL-R following single- and double-bundle ACL-R [35]. Additionally, a recent study from the NZACL investigated the effect of suspensory versus tibial fixation devices on revision rates following ACL-R. Moreover, data from Kaiser Permanente’s ACLR registry was recently used to identify differences in revision risk with respect to the timing of ACL-R [36] and concomitant meniscus surgery [37]. Importantly, data collection from non-operatively treated patients with ACL injury enables the comparison of outcomes following operative and non-operative treatment approaches [38, 39].

The previous comparative studies highlight some of the ways registry data may be applied to answer questions about the influence of treatment variables on patient outcomes following ACL-R. The inclusion of additional treatment-related factors and a growing interest in ACL-R augmentation with lateral extraarticular tenodesis (LET) are likely to lead to more comparative registry-based studies in the coming years.

### Predictors of Clinical Outcomes and ACL-R Failure

One of the many advantages of prospectively collected registry data is the ability to use the dataset for identifying demographic, injury-related, and surgical predictors of outcomes following ACL-R. Recent predictor studies [40–44] harnessed statistical methods, including but not limited to multivariable logistic and linear regressions, Kaplan-Meier survival curves, and Cox proportional hazards models to predict the influence of treatment variables on superior or inferior PROMs and assess the risk of revision ACL surgery over time, respectively, while aiming to account for the complex interaction between a large number of factors. Also, a couple of registry-based studies have already harnessed the use of artificial intelligence (AI) and machine learning (ML) technology in order to predict outcomes following treatment of ACL injury [45•, 46•]. With the emerging role of AI and ML, registry data will likely enable the development of algorithms designed to predict the individualized risk of ACL-R failure and inferior patient outcomes based on treatment variables [45•, 46•].

### Novel Applications of Registry Data

Perhaps one of the key arguments advocating the importance of registry data is the versatility of novel applications published over the recent years. Registry data has been used to define the minimal important change (MIC) of the KOOS in patients undergoing ACL-R, using an anchor-based questionnaire and receiver operating characteristic (ROC) curves [47]. Moreover, following the definition of patient acceptable symptom state (PASS) for patients undergoing ACL-R [48], registry studies were able to investigate factors influencing the achievement of PASS at different follow-up intervals [49, 50].

While numerous studies from the Scandinavian ACL registries report statistically significant findings, a subset of queried registry data may not always be as robust as the parent dataset. Recent assessment of the statistical robustness of data from Denmark, Norway, and Sweden using the Fragility Index (FI) method, measuring the fragility of the reported study results, revealed that the robustness of statistically significant results displays a large variation and warrants cautious interpretation [51•].

Finally, the incorporation of data from non-operatively treated ACL-injured patients, rehabilitation-specific registries assessing psychometric PROMs, measures of risk appraisal, and muscle function [52, 53] will open new avenues for the evaluation of ACL injury treatment outcomes. Merger of data from registries with different aims will provide a more complete picture of the operative and postoperative course of ACL-R patients and permit more complex analyses of outcomes following ACL surgery.

## Strengths and Limitations with Registry Data

Several strengths and limitations have previously been associated with registry-based research. The ACL registries include a large amount of data including detailed information on treatment- and patient-related factors as well as outcome measures allowing the possibility for a great overview of the epidemiology of ACL injuries [19, 54]. Additionally, large amounts of available data have been allowing an opportunity for less time-consuming studies, making registry-based studies more practical and easier to perform [55]. Nonetheless, the registry-based research carries some limitations. The compliance with regard to reporting of PROMs can be difficult to achieve with these registries, and there is a constant need for balance between the response rate of the patients and the amount of data that can be collected [55]. Moreover, the collected data and follow-up times for each individual ACL registry are not identical allowing challenges for combining and comparing of data from different registries and subsequently making generalization of the study results more difficult [31]. Furthermore, most of the ACL registries are still using KOOS as a primary outcome, which has been previously reported to be an “inappropriate” PROM for ACL injuries [31, 56, 57]. Thus, the KOOS has been reported to have a lack of responsiveness as well as including irrelevant content such as certain items in the subscales: pain and activities of daily living [58••, 59]. Consequently, the use of inappropriate PROMs can lead to false negative results and potentially misleading conclusions [55, 57, 58••, 60]. Lastly, today most of the registries focus primarily on collecting data on surgically treated patients making comparison between the non-operatively and operatively treated populations difficult.

## Future of ACL Research Based on Registry Data

High-quality registry data plays a crucial role in assessing the outcomes of ACL-R at the level of the national population. Suggestions to improve the future utility of registry data include recruitment of non-operatively treated patients with ACL injury, the conduction of registry-based RCTs, the use of validated PROMs specific to ACL injury, and international collaborations between existing and newly established ACL registries (Table 3) [31, 61].

**Table 3:** Take-home messages

Clinical insight gained from robust registry data plays a crucial role in research uncovering the interplay between the epidemiology, risk factors of injury, surgical variables, and their effect on clinical and functional patient outcomes.	
While the availability of comprehensive demographic, injury-related, surgical, and patient-reported outcome data contributed to a variety of high-quality research pertaining to the surgical treatment of ACL tears, the future goal of ACL-R registries should be to improve the specificity of PROMs, coverage of patients over time, and granularity of the collected data.	
International collaboration between registries has the potential to provide a dataset large enough for the prediction of patient outcomes using ML algorithms and pave the way for multicenter registry-based RCTs.	

*ACL* anterior cruciate ligament, *ACL-R* anterior cruciate ligament reconstruction, *ML* machine learning, *PROMS* patient-reported outcome measures, *RCTs* randomized controlled trials

Future registry studies will likely implement the use of AI and ML algorithms to analyze large volumes of patient data, develop clinical decision-making tools, and assess the patient-specific risk of inferior treatment outcomes. Currently, only a couple of registry-based studies harnessed the use of AI and ML technology [45•, 46•], but international collaboration and an increasing volume of data will likely lead to an increase in the popularity of this approach. While AI and ML provide attractive novel approaches to study ACL-R outcomes, it is essential to encourage the development of clinically meaningful models, which augment the surgical decision-making process and translate well to everyday clinical use. An additional future challenge faced by ACL registries is the need for standardization of collected data. This step is essential, not only to streamline collaboration between multiple registries, but also to facilitate the interpretation of results from an increasing volume of registry-based studies on ACL-R. While recent efforts to develop crosswalks between frequently used PROMs in ACL research reported success on a group level [62], the ultimate solution will be to establish a consensus regarding the use of injury-specific outcome measures for registry-based ACL research.

## Conclusion

The ACL registries have had an important role in increasing our understanding of ACL injuries and their treatment, and subsequently, provided increased knowledge on PROMs followed by ACL-R. However, the current ACL registries are also facing some challenges, pressing the need for new methods and techniques to be implemented to ensure further good quality and clinical applicability of study findings. There is a need for standardization of the collected data between the registries, with the future of registry-based ACL-R research having the potential to use AI and ML to create clinical decision-making tools and analyzing outcomes following ACL-R. Continuous development of the ACL registries and future studies are warranted to expand our understanding and improve the treatment of ACL injuries.
